# Evidence That Breast Tissue Stiffness Is Associated with Risk of Breast Cancer

**DOI:** 10.1371/journal.pone.0100937

**Published:** 2014-07-10

**Authors:** Norman F. Boyd, Qing Li, Olga Melnichouk, Ella Huszti, Lisa J. Martin, Anoma Gunasekara, Gord Mawdsley, Martin J. Yaffe, Salomon Minkin

**Affiliations:** 1 Campbell Family Institute for Breast Cancer Research, Ontario Cancer Institute, Toronto, Ontario, Canada; 2 Imaging Research, Sunnybrook Health Sciences Centre, Toronto, Ontario, Canada; Geisel School of Medicine at Dartmouth, United States of America

## Abstract

**Background:**

Evidence from animal models shows that tissue stiffness increases the invasion and progression of cancers, including mammary cancer. We here use measurements of the volume and the projected area of the compressed breast during mammography to derive estimates of breast tissue stiffness and examine the relationship of stiffness to risk of breast cancer.

**Methods:**

Mammograms were used to measure the volume and projected areas of total and radiologically dense breast tissue in the unaffected breasts of 362 women with newly diagnosed breast cancer (cases) and 656 women of the same age who did not have breast cancer (controls). Measures of breast tissue volume and the projected area of the compressed breast during mammography were used to calculate the deformation of the breast during compression and, with the recorded compression force, to estimate the stiffness of breast tissue. Stiffness was compared in cases and controls, and associations with breast cancer risk examined after adjustment for other risk factors.

**Results:**

After adjustment for percent mammographic density by area measurements, and other risk factors, our estimate of breast tissue stiffness was significantly associated with breast cancer (odds ratio = 1.21, 95% confidence interval = 1.03, 1.43, p = 0.02) and improved breast cancer risk prediction in models with percent mammographic density, by both area and volume measurements.

**Conclusion:**

An estimate of breast tissue stiffness was associated with breast cancer risk and improved risk prediction based on mammographic measures and other risk factors. Stiffness may provide an additional mechanism by which breast tissue composition is associated with risk of breast cancer and merits examination using more direct methods of measurement.

## Introduction

Physical forces generated by interactions between cells, and between cells and the extracellular matrix, influence a variety of cell functions including cell growth, survival, motility and differentiation [1]. These forces are associated with an increase in tissue stiffness. Breast cancer is characterized by increasing stiffness of breast tissue that contributes to the detection of disease by palpation or by elastography. Kass et al have suggested that the mechanical properties of the tissue might also influence breast cancer risk [2].

Radiologically dense breast tissue on mammography, referred to as mammographic density, reflects variations in breast tissue composition. Epithelial and stromal tissues attenuate x-rays more than fat and appear dense or white, while fat is more radiolucent and appears dark. Compared to women with little or no density, those with extensive density have a 4–6 fold greater risk of developing breast cancer [3], [4]. Extensive mammographic density is associated with both an increased number of cells and extensive collagen [5] and also with increased proteoglycan expression [6], all factors that may increase breast tissue stiffness. It is known that a quantitative classification of breast tissue composition is associated with risk of breast cancer. We here examine the possibility that the biomechanical properties of breast tissue are also associated with risk of the disease [2]. We use a set of idealized assumptions about the shape of the breast to obtain preliminary estimates of the biomechanical properties of breast tissue and their relation to breast cancer risk.

We have used measurements of the breast made in a case-control study of mammographic density and risk of breast cancer to estimate the extent to which the breast is deformed during compression and derive an estimate of the stiffness of breast tissue. We have compared the estimate of stiffness in cases and controls after adjustment for other breast cancer risk factors.

## Methods

Details of the recruitment of subjects and of their characteristics, and of the methods used to measure breast tissue volume and area, have been given elsewhere [7] and will be summarized briefly here.

### A. Ethics statement

Written informed consent was obtained from all subjects who authorized the release of their mammograms for the purpose of density measurement and agreed to take part in a telephone interview that asked about factors related to breast cancer risk. Ethics approval for the study was obtained from the University Health Network, Mount Sinai Hospital, Sunnybrook and Women’s College Hospital and from Cancer Care Ontario (for the Ontario Breast Screening Programme).

### B. Recruitment of subjects

We have recruited cases and controls that had been examined on mammography units in the clinics of Mount Sinai Hospital, Women’s College Hospital, University Health Network, Sunnybrook Health Sciences Centre, and the North York and Scarborough sites of the Ontario Breast Screening Programme (OBSP), all in Toronto, Canada. The selection of cases and controls was from all subjects having mammography in these sites during the period of 13 March, 2000 and 7 July, 2003. All mammography units in these clinics were calibrated using the methods described below. All subjects examined in OBSP sites were seen for screening mammography, while those seen in hospital sites are likely to have included some for screening and some for evaluation of symptomatic breast disease. OBSP screening sites contributed only 8.2% of the cases and control subjects. The number of cases was small compared to hospital sites and only 1 control could be matched per case.

#### 1. Identification and selection of cases

Potentially eligible cases were all incident cases diagnosed between 13 March, 2000 and 7 July, 2003 in hospitals where the machines had been calibrated and with at least one screen-film mammogram performed before diagnosis. Cases with bilateral synchronous breast cancer, in which a screen-film mammogram without radiological signs of cancer was not available, were excluded. Subjects who had breast implants, or reduction mammoplasty were also excluded.

#### 2. Identification and selection of controls

Controls were selected from the same study population as cases. We attempted to identify 2 controls for each case, one examined on the same mammography machine as the case and the other from a different machine. However, some mammography clinics had only one machine and for these we recruited only controls examined on the same machine as the cases. The two types of controls were combined for this analysis.

#### 3. Recruitment and data collection

With the agreement of their physician, potentially eligible case and control subjects were contacted by mail, the study explained and they were asked for consent to the use of their mammogram and to participate in a telephone interview to provide information about risk factors for breast cancer.

### C. Measurement of mammographic density

After consent had been obtained, screen-film mammograms for the case and control subjects selected were obtained from the participating mammography units. To “blind” the process of measurement to case or control status, we selected the image of the breast contra-lateral to the cancer, and the corresponding mammograms in the matched controls. Two methods of measurement that have been described previously [8], [9] were applied to the cranial-caudal view mammograms.

#### 1. Measurement of breast area

Computer-assisted measurement of mammographic density was carried out by one reader (NFB) using *Cumulus 4* software. Measurements of the areas of dense tissue and total area were generated and percent density calculated.

#### 2. Measurement of breast tissue volumes

Each mammography machine from which we recruited was calibrated to determine the relationship between the image signal (optical density or blackness of the processed film value) in each pixel, the exposure factors (kilovoltage, milliamp seconds (mAs), tube target and beam filter) and the amount of radiation transmitted by the breast. The latter can then be related to the combination of breast thickness and composition by imaging a “phantom” composed of steps of tissue-equivalent plastics of different thicknesses and representing a range of combinations of fat and fibroglandular tissue [9]. Therefore, under specified exposure conditions, for a given measured image signal the tissue composition corresponding to each pixel can be estimated from the screen-film mammogram if the breast thickness is known. The total volume of dense (fibroglandular) tissue was obtained by multiplying the fibroglandular fraction for each pixel by the area of the pixel and the thickness of the compressed breast at that location and then summing over all pixels. Similarly, the total breast volume was simply the sum of the areas of all pixels in the image of the breast, each multiplied by the corresponding breast thickness.

Compressed breast thickness is the distance between the compression paddles of a mammography machine and the breast supporting tabletop when the mammogram is obtained. Breast thickness is not constant across the breast area; and we generated a thickness map for each x-ray image to calculate the total volume and dense volume of the breast. Equations to predict a thickness map for each image were developed from the readout thickness reported by each mammography machine, coordinates in the plane parallel to the breast support table, and the compression force reported by a mammography machine.

#### 3. Estimation of breast tissue stiffness

**Figure 1:**
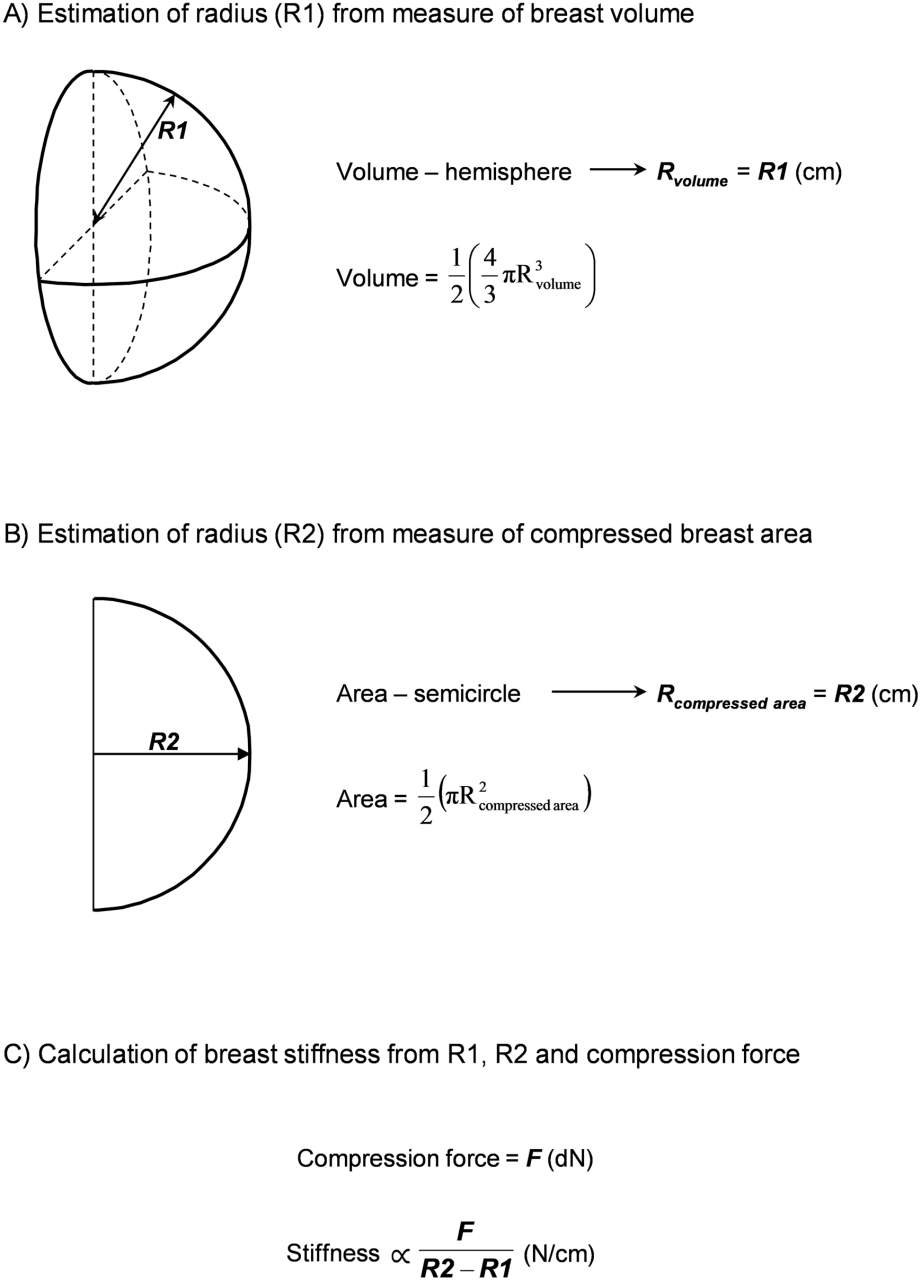
Estimation of breast stiffness. A. Estimation of radius (R1) from measure of breast volume B. Estimation of radius (R2) from measure of compressed breast area C. Calculation of breast stiffness from R1, R2 and compression force.

Figure 1 shows how breast tissue stiffness was estimated from the measures of volume and area described above by making three idealized assumptions. First we assumed that the measured breast volumes and the projected area of the compressed breast were true measures of these entities. We further assumed that the shape of the uncompressed breast could be represented by a hemisphere and that of the mammographic area by a semicircle. The measured volume of the breast remains unchanged regardless of the shape it is assumed to occupy. The assumption that the shape of the uncompressed breast is a hemisphere allows us to calculate the radius of the hemisphere to compare with the radius of the compressed breast obtained from the mammogram. In the absence of compression the projected area of the breast in a mammogram is expected to be equal to the area of a section of the hemisphere and to have the same radius.

We further assume that the contralateral breast in cases is representative of the subject. It is known that breast tissue composition, assessed by either mammography [10], [11] or by measured breast water using magnetic resonance [12] is highly symmetrical. There has however not been to date any assessment of the symmetry of the biomechanical properties of breast tissue.

We defined the difference between the radius of the mammographic area semicircle, and the radius of the volumetric hemisphere as “deformation”. With the compression force recorded with each mammogram the measure of deformation was used to calculate “stiffness” by the formula: Force/Deformation (N/cm), where N denotes deca-Newtons and cm centimetres.

### D. Statistical methods

All subjects with available volumetric breast measurements had deformation computed. We excluded from the analysis two subjects with deformation smaller than zero. For selected characteristics of the case and control subjects, we calculated mean and standard deviation (SD) for continuous variables, and proportion for categorical variables. Differences between cases and controls were ascertained by t-test for symmetrically distributed continuous variables, Wilcoxon rank-sum test for the non-symmetrical ones, and chi-square test for categorical variables.

We used linear regression models to examine the association between stiffness and breast cancer status (after adjusting for other risk factors for breast cancer such as age at mammogram, age at birth of first child, weight, height, menopausal status (pre/post), and parity (parous/nonparous), with and without adjustment for breast density measurements. We applied natural log transformation to stiffness, square root transformation to all measurements of breast area, and cube root transformation to all measurements of breast volume to make the distributions more symmetrical with stable variance.

We used logistic regression modeling to examine the association of the area and volume measurements with risk of breast cancer before and after adjustment for deformation or stiffness, in addition to adjustment for the risk factors for breast cancer mentioned above. All p-values were calculated from two-tailed tests of statistical significance.

We imputed the mean value of weight for three subjects, menopausal status (post) for two subjects, and the mean value of age at birth of first child for 280 subjects. For fourteen subjects with force recorded as zero, we used the mid-point value (20) between the machine recording threshold (30) and the minimum force required to produce pressure (10). The results obtained using this imputation were very similar to those from the analysis excluding these 14 subjects. All statistical analyses were carried out using Statistical Analysis Systems (SAS) 9.2 software.

## Results

### A. Characteristics of subjects

Table 1 shows selected characteristics of the case and control subjects. The average age of the subjects studied was 59 years, and most were parous and postmenopausal. Cases had a slightly later age at menopause than controls but the distributions of other risk factors for breast cancer were similar in cases and controls. Table 2 shows average measures of mammographic density by both area and volume. The area and volume of dense tissue were both greater in cases than in controls. Average measures of total area and volume were both smaller in cases than in controls. The average interval between the date of mammography and the date of interview for the study was for 20 months for controls (SD: 8 months) and 9.6 months for cases (SD: 4 months).

**Table 1:** Risk factors by case-control status.

	Mean (SD) or %		*P* a
	Cases (*n* = 362)	Controls (*n* = 656)	
Height (cm)	162.6 (6.9)	163.2 (6.4)	0.13 (0.05^W^)
Weight (kg), *n* = 360, 655	68.4 (14.3)	68.1 (14.6)	0.75
Body mass index (kg/m^2^), *n* = 360, 655	25.9 (5.2)	25.6 (5.4)	0.35
Age at mammogram (years)	59.7 (11.0)	59.0 (11.0)	0.37
Age at menarche (years), *n* = 359, 654	12.7 (1.4)	12.8 (1.5)	0.65
Parity (% parous)	71.3	73.2	0.52
Age at birth of first child (years), *n* = 258, 479	26.3 (5.0)	26.6 (5.5)	0.60
Number of live births in parous women, *n* = 258, 480	2.3 (1.0)	2.3 (1.1)	0.98
Menopausal status (% post), *n* = 361, 655	68.4	69.8	0.66
Age at menopause (years), *n* = 247, 491	49.0 (6.1)	47.8 (6.3)	0.02
HRT^b^ ever used (% yes), *n* = 362, 655	45.0	45.0	0.997
Years HRT^b^ used (years) in everuser, *n* = 163, 295	8.8 (7.9)	8.9 (8.7)	0.38
Family history^c^ (% yes), *n* = 359, 653	21.7	24.8	0.27

a *P* is a p-value from a two-sided two-sample t-test for symmetrically distributed continuous variables or Wilcoxon rank-sum test for non-symmetrically distributed continuous variables and chi-square test for categorical variables.

b Hormone replacement therapy.

c First degree relatives with breast cancer.

**Table 2:** Breast measurements by case-control status.

	Mean (SD)		*P* a
	Cases (*n* = 362)	Controls (*n* = 656)	
Compression force (N)^b^	104.9 (32.0)	103.1 (31.5)	0.52^c^
**Area breast measurements**			
Percent dense area	33.1 (20.5)	30.2 (19.8)	0.04
Dense area (cm^2^)	40.9 (26.9)	37.5 (25.6)	0.05
Non-dense area (cm^2^)	101.2 (64.2)	108.1 (67.7)	0.11
Total area (cm^2^)	142.1 (60.7)	145.6 (63.8)	0.44
**Volume breast measurements**			
Percent dense volume	11.3 (16.1)	8.9 (13.9)	0.009
Dense volume (cm^3^)	58.1 (76.7)	47.0 (76.1)	0.005
Non-dense volume (cm^3^)	669.6 (375.8)	710.7 (420.4)	0.23
Total volume (cm^3^)	727.7 (360.2)	757.6 (412.2)	0.51

a *P* is a p-value from a two-sided two-sample t-test, based on transformed variables. Area breast measurements were square root transformed and volume breast measurements were cubic root transformed for the analysis. Mean and standard deviation were calculated using untransformed data.

b 14 subjects with compression force under minimum detectable threshold was imputed as half of the minimum detectable value. Mean and standard deviation were calculated based on imputed variable.

c P-value from Wilcoxon rank-sum test.

### B. Association of stiffness with risk of breast cancer

**Figure 2:**
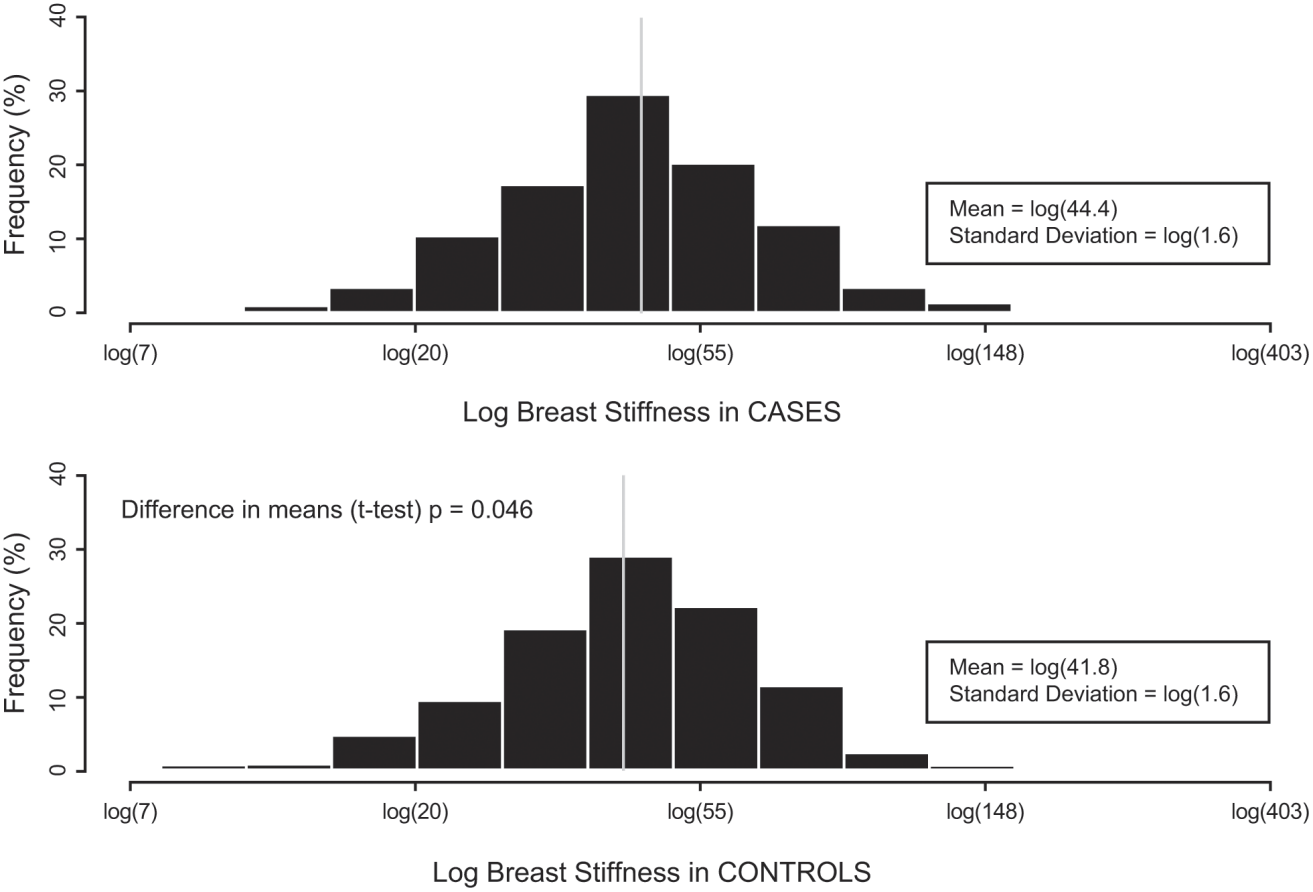
Histograms of the distributions of the stiffness measures in cases and controls. The stiffness measures were natural logarithm transformed. In each plot, the thin vertical line represents the mean of the distribution.

Figure 2 shows histograms of the unadjusted distributions of the natural logarithm transformed stiffness measures in cases (mean = log (44.4), standard deviation (SD) = log (1.6)) and controls (mean = log (41.8), SD = log (1.6)), (p = 0.046).

**Figure 3:**
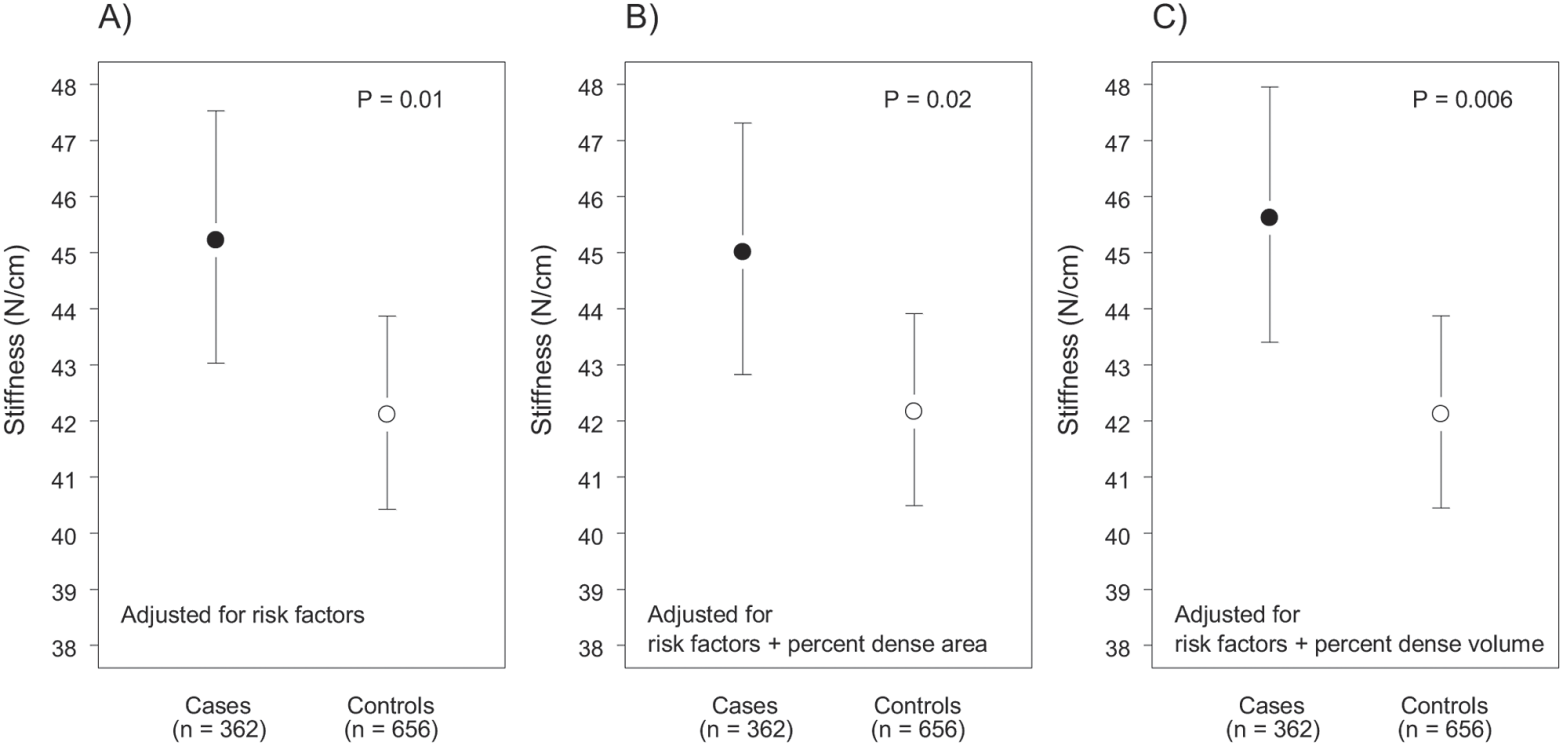
Least square means of stiffness in cases and controls, adjusted for risk factors. Risk factors include: age at mammogram (linear and quadratic terms), age at birth of first child, weight (kg), height (cm), menopausal status (pre/post) and parity (parous/nonparous). Stiffness (N/cm) was natural logarithm transformed in the analysis. The least square means shown are back transformed to the original scale. Bars show 95% confidence interval. P is the p-value for the significance of case control difference. When adjusted for percent dense area, square root transformation was used and model includes linear and quadratic terms. When adjusted for percent dense volume, cubic root transformation was used and model includes linear and quadratic terms.

Figure 3A–C shows the least square means and 95% confidence interval for measures of stiffness in cases and controls, adjusted for the demographic and reproductive risk factors shown in the figure legend. The least square means of stiffness were 45.22 in cases (95% CI: 43.03, 47.53) and 42.11 (95% CI: 40.43, 43.87) in controls (p = 0.01). After additional adjustment for percent dense area (Figure 3B) the least square means of stiffness were 45.01 in cases (95% CI: 42.83, 47.31) and 42.17 (95% CI: 40.49, 43.91) in controls (p = 0.02). After adjustment for percent dense volume (Figure 3C) the least square means of stiffness were 45.62 in cases (95% CI: 43.40, 47.95) and 42.12 (95% CI: 40.45, 43.87) in controls (p = 0.006).

The ORs and 95% CIs for quartiles of stiffness with reference to quartile 1, were 0.94 (0.65, 1.36) for quartile 2, 1.17 (0.81, 1.70) for quartile 3, and 1.35 (0.91, 1.99) for quartile 4.

Table 3 shows the effects on breast cancer risk prediction of adding the measure of stiffness to models that included mammographic measures of percent density and dense tissue by either the volume or area methods. The volume and area measures of percent density were treated as continuous variables and respectively cube root and square root transformed in analysis. All analyses were carried out with adjustment for other risk factors (shown in the footnote to Table 3), and before and after the inclusion of the stiffness measures.

**Table 3:** Mammographic measures, stiffness and breast cancer risk.

	Breast Density Measure:		*Stiffness:*		Model
*Included in the Model:*	IQOR (95% CI)^b^	P-value^c^	IQOR (95% CI)^b^	P-value^c^	AUC^d^
Risk Factors^a^ (RF) and ***Stiffness***	-		1.24 (1.05, 1.46)	0.01	0.568
RF and **Percent dense area**	1.58 (1.22, 2.05)	0.0005	-		0.586
RF and **Percent dense area** and***Stiffness***	1.54 (1.19, 2.00)	0.001	1.21 (1.03, 1.43)	0.02	0.594
RF and **Dense area**	1.22 (1.04, 1.43)	0.01	-		0.568
RF and **Dense area** and ***Stiffness***	1.27 (1.08, 1.49)	0.004	1.29 (1.09, 1.52)	0.003	0.588
RF and **Percent dense volume**	1.42 (1.15, 1.75)	0.001	-		0.581
RF and **Percent dense volume** and***Stiffness***	1.46 (1.18, 1.81)	0.0004	1.27 (1.08, 1.51)	0.004	0.595
RF and **Dense volume**	1.34 (1.11, 1.62)	0.001	-		0.582
RF and **Dense volume** and ***Stiffness***	1.40 (1.16, 1.69)	0.0004	1.29 (1.09, 1.53)	0.004	0.598

N = 1018 (362 cases and 656 controls). Stiffness (N/cm) was log transformed. Square root transformation was applied on area measurements, cubic root transformation on volume measurements. Standardization was applied on transformed variables.

a Risk Factors (RF): age at mammogram, age at birth of first child, weight (kg), height (cm), menopausal status (pre/post) and parity (parous/nonparous).

b Inter-quartile odds ratios and 95% confidence intervals were calculated as.

IQOR = exp{beta*IQR}, where beta is the standardized regression coefficient, and IQR is the observed inter-quartile range, in controls, for standardized transformed percent dense area, percent dense volume, dense area, dense volume and stiffness: 1.51, 1.43, 1.15, 1.37 and 1.19, respectively.

c P-value corresponds to the change in the likelihood ratio for the addition of the specific variable to a model with all others included.

d AUC: area under the curve.

The interquartile odds ratio (IQOR) for stiffness, adjusted for other non-mammographic risk factors was 1.24 (95% confidence interval (CI): 1.05, 1.46), p = 0.01. After adjustment for percent mammographic dense area in addition to other risk factors, the IQOR for stiffness was 1.29 (95% CI: 1.09, 1.52), p = 0.003. After adjustment for percent dense volume, the IQOR for stiffness was 1.27 (95% CI: 1.08, 1.51), p = 0.004.

Before inclusion of the stiffness measure, percent mammographic density, by the area (p = 0.0005) and volume (p = 0.001) measures, and the area (p = 0.01) and volume (p = 0.001) of dense tissue were all significantly and positively associated with risk of breast cancer as separate predictors. After the inclusion in the model of the stiffness measure, the association of percent mammographic density by the area measure was slightly reduced, as shown by the regression coefficient and the interquartile odds ratio, although the area under receiver operating characteristic curve (AUC) increased by 1.4%. For all other mammographic measures the regression coefficients and interquartile odds ratios increased after the inclusion of stiffness, and the associated AUCs increased by between 2.4 and 3.5%.

## Discussion

These results show that estimates of the stiffness of breast tissue during compression are associated with risk of breast cancer after adjustment for other known risk factors including percent mammographic density, which is strongly associated with risk of breast cancer [13].

Strengths of the present study include the relatively large numbers of incident cases and matched controls, with breast images acquired prospectively from calibrated machines. However, stiffness was not measured directly but was based on idealized assumptions about the shape of the breast volume and projected area that are potentially subject to error. For example, calculation of the breast volume measures requires accurate information about the thickness of the compressed breast at each pixel in the image. Yaffe et al have shown elsewhere that the measured percent density by volume is very sensitive to small errors in the measurement of breast thickness [14], [15]. There is however no reason to expect errors in the measurement of thickness to lead to systematic under or over estimation of breast volumes, or to differ between cases and controls. An additional potential source of error is the compression force during mammography, that was used here in the calculation of stiffness and which may not be accurately calibrated. These potential sources of error in the measures used here are likely to lead to underestimation of associations with risk of breast cancer and suggest that direct measurement of breast tissue deformation and stiffness and of the compression force may further improve risk prediction. Further, our results are based on measurements obtained from screen-film mammography that has now largely been replaced by digital mammography in which density is presented in a manner different from film. However, as we note below, the further development and use of measured stiffness is more likely to depend on the application of ultrasound or magnetic resonance than on mammography.

Percent mammographic density, as assessed here in the area measurement, reflects variations in breast tissue composition [5] and has repeatedly been shown to be strongly associated with risk of breast cancer, with 4–5 fold differences in risk of the disease between women with more than 75% percent density compared to those with less than 10% [13], [3]. The smaller estimates of risk seen here, and the associated results of receiver operating characteristic analysis, may be attributable to the use of quartiles rather than the aforementioned categories, and the methods used to recruit subjects. Our method of recruitment selected for breast cancers detected by mammography, where the gradient in risk associated with density is less than for all breast cancers [3].

There are abundant data to suggest that an association between breast tissue stiffness and breast cancer risk is biologically plausible. Epithelial and stromal cells, collagen, and fat, the tissue components that contribute to variations in mammographic density, are related to each other in several ways. Epithelial and stromal cells communicate with each other by means of paracrine growth factors [16]. Collagen is a product of stromal fibroblasts, and adipocytes develop from the differentiation of stromal preadipocytes [17]. Factors that affect one of these components may, therefore, affect the others, either directly or indirectly, and each component has properties that may influence the risk of progression of breast cancer.

Collagen and the stromal matrix are products of stromal cells that may, through their mechanical and other properties, facilitate tumor invasion [18]. Interactions between stroma and epithelium are also known to influence breast development and the changes in breast structure that take place during, pregnancy, lactation, and involution and during tumorigenesis [19], [20]. The extracellular matrix, which comprises collagens, fibronectin, laminins, polysaccharides, and proteoglycans, plays a key role in these processes, and there is a large and rapidly growing literature on the molecules that mediate the influence of the extracellular matrix on the greater stiffness of stroma associated with breast cancer compared to normal breast tissue (see [1], [21], [22] for reviews).

To date, there has been limited application of these basic science findings to understanding the association between mammographic density and risk of breast cancer. In addition to having greater amounts of collagen, epithelial and stromal cells, and larger areas that are immunohistochemically positive for Insulin-like growth factor-I (IGF-1), radiologically dense breast tissue also has greater amounts of the stromal matrix regulatory protein tissue inhibitor metalloproteinase-3 [23]. Metalloproteinases that regulate stromal matrix can also regulate the activation of growth factors and influence susceptibility to breast cancer [2]. Expression of the proteoglycans lumican and decorin has been found to be increased in stromal tissue associated with breast cancer and, in the absence of cancer, in women with extensive mammographic density [6]. Proteoglycans bind growth factors, contribute to the mechanical integrity of tissues, may influence the stiffness of breast tissue, and modify tissue behavior [1].

Our results suggest that knowledge of both the quantity and stiffness of breast tissue may improve prediction of breast cancer risk in individuals, and facilitate research into the tissue factors that influence breast cancer risk. Stiffness may provide an additional mechanism by which breast tissue composition influences risk of breast cancer and merits examination using more direct methods of measurement such as elastography using ultrasound or magnetic resonance [24], [25], [26]. Further, stronger study designs such as cohort studies with prolonged follow-up preceding the diagnosis of breast cancer, that would allow assessment of the predictive value of stiffness and rule out the possibility that this is a consequence of cancer, would be particularly valuable.

## Conclusions

An estimate of breast tissue stiffness was associated with breast cancer risk, and improved risk prediction based on mammographic measures and other risk factors. Stiffness may provide an additional mechanism by which breast tissue composition is associated with risk of breast cancer and merits examination using more direct methods of measurement.

## References

[1] Butcher DT, Alliston T, Weaver VM (2009) A tense situation: forcing tumour progression. Nat Rev Cancer 9: 108–122. 19165226 10.1038/nrc2544 PMC2649117

[2] Kass L, Erler JT, Dembo M, Weaver VM (2007) Mammary epithelial cell: Influence of extracellular matrix composition and organization during development and tumorigenesis. Int J Biochem Cell Biol 39: 1987–1994. 17719831 10.1016/j.biocel.2007.06.025 PMC2658720

[3] Boyd NF, Guo H, Martin LJ, Sun L, Stone J, et al (2007) Mammographic density and the risk and detection of breast cancer. N Engl J Med 356: 227–236. 17229950 10.1056/NEJMoa062790

[4] Boyd NF, Martin LJ, Bronskill MJ, Yaffe MJ, Duric N, et al (2010) Breast tissue composition and susceptibility to breast cancer. J Natl Cancer Inst 102: 1224–1237. 20616353 10.1093/jnci/djq239 PMC2923218

[5] Li T, Sun L, Miller N, Nicklee T, Woo J, et al (2005) The association of measured breast tissue characteristics with mammographic density and other risk factors for breast cancer. Cancer Epidemiol Biomarkers Prev 14: 343–349. 15734956 10.1158/1055-9965.EPI-04-0490

[6] Alowami S, Troup S, Al-Haddad S, Kirkpatrick I, Watson PH (2003) Mammographic density is related to stroma and stromal proteoglycan expression. Breast Cancer Res 5: R129–R135. 12927043 10.1186/bcr622 PMC314426

[7] Boyd NF, Martin LJ, Gunasekara A, Melnichouk O, Maudsley G, et al (2009) Mammographic density and breast cancer risk: evaluation of a novel method of measuring breast tissue volumes. Cancer Epidemiol Biomarkers Prev 18: 1754–1762. 19505909 10.1158/1055-9965.EPI-09-0107

[8] Byng JW, Boyd NF, Fishell E, Jong RA, Yaffe MJ (1994) The quantitative analysis of mammographic densities. Phys Med Biol 39: 1629–1638. 15551535 10.1088/0031-9155/39/10/008

[9] Pawluczyk O, Augustine BJ, Yaffe MJ, Rico D, Yang J, et al (2003) A volumetric method for estimation of breast density on digitized screen-film mammograms. Med Phys 30: 352–364. 12674236 10.1118/1.1539038

[10] Byng JW, Boyd NF, Little L (1996) Symmetry of projection in the quantitative analysis of mammographic images. Eur J Cancer Prev 5: 319–327. 8972250 10.1097/00008469-199610000-00003

[11] Vachon CM, Brandt KR, Ghosh K, Scott CG, Maloney SD, et al (2007) Mammographic breast density as a general marker of breast cancer risk. Cancer Epidemiol Biomarkers Prev 16: 43–49. 17220330 10.1158/1055-9965.EPI-06-0738

[12] Hennessey S, Huszti E, Gunasekara A, Salleh A, Martin L, et al (2014) Bilateral symmetry of breast tissue composition by magnetic resonance in young women and adults. Cancer Causes Control 25: 491–497. 24477331 10.1007/s10552-014-0351-0 PMC3942631

[13] McCormack VA, dos Santos Silva I (2006) Breast density and parenchymal patterns as markers of breast cancer risk: A meta-analysis. Cancer Epidemiol Biomarkers Prev 15: 1159–1169. 16775176 10.1158/1055-9965.EPI-06-0034

[14] Yaffe MJ, Boone JM, Packard N, Alonzo-Proulx O, Huang S-Y, et al (2009) The myth of the 50-50 breast. Med Phys 36: 5437–5443. 20095256 10.1118/1.3250863 PMC2787062

[15] Pawluczyk O, Yaffe MJ (2001) Field nonuniformity correction for quantitative analysis of digitized mammograms. Med Phys 28: 438–444. 11339739 10.1118/1.1359244

[16] Hagios C, Lochter A, Bissell M (1998) Tissue architecture: the ultimate regulator of epithelial function? Royal Society 353: 857–870. 10.1098/rstb.1998.0250 PMC1692274 9684283

[17] Zangani D, Darcy KM, Masso-Welch PA, Bellamy ES, Desole MS, et al (1999) Multiple differentiation pathways of rat mammary stromal cells *in vitro*: acquisition of a fibroblast, adipocyte or endothelial phenotype is dependent on hormonal and extracellular matrix stimulation. Differentiation 64: 91–101. 10234806 10.1046/j.1432-0436.1999.6420091.x

[18] Provenzano PP, Inman DR, Eliceiri KW, Knittel JG, Yan L, et al (2008) Collagen density promotes mammary tumor initiation and progression. BMC Med 6: 11. 18442412 10.1186/1741-7015-6-11 PMC2386807

[19] Wiseman BS, Werb Z (2002) Stromal effects on mammary gland development and breast cancer. Science 296: 1046–1049. 12004111 10.1126/science.1067431 PMC2788989

[20] Nelson CM, Bissell MJ (2006) Of extracellular matrix, scaffolds, and signaling: tissue architecture regulates development, homeostasis, and cancer. Annu Rev Cell Dev Biol 22: 287–309. 16824016 10.1146/annurev.cellbio.22.010305.104315 PMC2933192

[21] Paszek MJ, Weaver VM (2004) The tension mounts: mechanics meets morphogenesis and malignancy. J Mammary Gland Biol Neoplasia 9: 325–342. 15838603 10.1007/s10911-004-1404-x

[22] Lu P, Weaver VM, Werb Z (2012) The extracellualr matrix: A dynamic niche in cancer progression. J Cell Biol 196: 395–406. 22351925 10.1083/jcb.201102147 PMC3283993

[23] Guo YP, Martin LJ, Hanna W, Banerjee D, Miller N, et al (2001) Growth factors and stromal matrix proteins associated with mammographic densities. Cancer Epidemiol Biomarkers Prev 10: 243–248. 11303594

[24] Wojcinski S, Boehme E, Farrokh A, Soergel P, Degenhardt F, et al (2013) Ultrasound real-time elastrography can predict malignancy in BI-RADS@-US 3 lesions. BMC Cancer. 13: 159. 10.1186/1471-2407-13-159 PMC3618252 23530903

[25] Wojcinski S, Dupont J, Schmidt W, Cassel M, Hillemanns P (2012) Real-time ultrasound elastography in 180 axillary lymph nodes: elasticity distribution in healthy lymph nodes and prediction of breast cancer metastases. BMC Med Imaging 12: 35. 23253859 10.1186/1471-2342-12-35 PMC3536617

[26] Lorenzen J, Sinkus R, Lorenzen M, Dargatz M, Leussler C, et al (2002) MR elastography of the breast: preliminary clinical results. rofo 174: 830–834. 12101471 10.1055/s-2002-32690

